# Trends in antimicrobial resistance in *Neisseria gonorrhoeae* in Hanoi, Vietnam, 2017–2019

**DOI:** 10.1186/s12879-020-05532-3

**Published:** 2020-11-05

**Authors:** Paul C. Adamson, Hung Van Le, Hai Ha Long Le, Giang Minh Le, Trung Vu Nguyen, Jeffrey D. Klausner

**Affiliations:** 1 Division of Infectious Diseases, David Geffen School of Medicine at UCLA, 10833 Le Conte Avenue CHS 52-215, Los Angeles, CA USA; 2 National Hospital of Venereology and Dermatology, Hanoi, Vietnam; 3 Hanoi Medical University, Hanoi, Vietnam; 4 National Hospital for Tropical Diseases, Hanoi, Vietnam; 5 Department of Epidemiology, Fielding School of Public Health, UC Los Angeles, Los Angeles, CA USA

**Keywords:** *Neisseria gonorrhoeae*, Gonorrhea, Ceftriaxone, Azithromycin, Drug resistance, bacterial, Anti-bacterial agents

## Abstract

**Background:**

Antimicrobial resistance (AMR) in *Neisseria gonorrhoeae* is an emerging global health threat. Surveillance of AMR in *N. gonorrhoeae* in the Western Pacific Region is important, as resistant strains have typically emerged from this region. There are sparse data regarding antibiotic susceptibility of *N. gonorrhoeae* from Vietnam. This study aimed to provide updated data on antibiotic susceptibilities in *N. gonorrhoeae* isolates from Hanoi, Vietnam.

**Methods:**

From 2017 to 2019, 409 *N. gonorrhoeae* clinical isolates were collected at the National Hospital for Venereology and Dermatology in Hanoi, Vietnam. Antibiotic susceptibility testing was performed by disk diffusion method according to the Clinical and Laboratory Standards Institute (CLSI) protocol. The zone diameters of inhibition were recorded and interpreted according to standard CLSI criteria, except for azithromycin, due to the absence of CLSI interpretation. Categorical variables were analyzed by Chi-square and Fisher’s exact tests. Linear regression was used to evaluate zones of inhibition by year.

**Results:**

Among the 409 isolates, no isolates were susceptible to penicillin, 98.3% were resistant to ciprofloxacin, and all isolates were susceptible to spectinomycin. There were 122/407 (30.0%) isolates resistant to azithromycin and there was an association between resistance and year (*p* <  0.01), ranging from 15.3% of isolates in 2017 to 46.7% of the isolates in 2018. Resistance to cefixime was found in 13/406 (3.2%) of isolates and there was no association by year (*p* = 0.30). Resistance to ceftriaxone occurred in 3/408 (0.7%) of isolates. Linear regression indicated the zone of inhibition diameters decreased by 0.83 mm each year for ceftriaxone (95% CI: − 1.3, − 0.4; *p* <  0.01) and decreased by 0.83 mm each year (95% CI: − 1.33, − 0.33; *p* <  0.01) for azithromycin; the association was not significant for cefixime (*p* = 0.07).

**Conclusions:**

We found decreasing susceptibility of *N. gonorrhoeae* to ceftriaxone and azithromycin, as well as a high prevalence of resistance to azithromycin, among isolates in Hanoi, Vietnam from 2017 to 2019. The trends of decreasing susceptibility to first-line treatments are concerning and highlight the urgency of addressing antimicrobial resistance in *N. gonorrhoeae*. Expanded surveillance efforts within the Western Pacific Region are critical to monitoring trends and informing treatment guidelines.

## Background

Antimicrobial resistance (AMR) in *Neisseria gonorrhoeae* is an emerging global health threat [1]. The World Health Organization (WHO) lists antibiotic-resistant *N. gonorrhoeae* as a high-priority pathogen and the U.S. Centers for Disease Control and Prevention (CDC) classifies antibiotic resistant *N. gonorrhoeae* as an urgent public health threat in the United States [2, 3].

*N. gonorrhoeae* has developed resistance to every class of antibiotics used for treatment [4]. Currently, dual treatment with azithromycin and ceftriaxone is widely recommended, although higher doses of ceftriaxone are being used as monotherapy in some settings [5–9]. Recently, strains with resistance to both ceftriaxone and azithromycin have been identified, likely originating from the Western Pacific Region (WPR) [10].

Surveillance of AMR in *N. gonorrhoeae* in the WPR is important, as resistant strains have typically emerged from this region [4]. The most recent report by the WHO Gonococcal Antimicrobial Surveillance Programme (GASP), including isolates through 2016, found that many countries in the WPR exceeded the 5% resistance thresholds to ceftriaxone and azithromycin that were historically used by the WHO to guide treatment recommendations [11]. Data regarding *N. gonorrhoeae* susceptibility to ceftriaxone and azithromycin in Vietnam is fairly limited, without reported data since 2016 [12, 13]. Here, we describe trends in antibiotic resistance in *N. gonorrhoeae* from 2017 to 2019 in Hanoi, Vietnam.

## Methods

From 2017 to 2019, *N. gonorrhoeae* bacterial isolates were collected from clinical specimens processed as part of routine clinical care at the National Hospital for Venereology and Dermatology in Hanoi, Vietnam. The bacterial isolates underwent antibiotic susceptibility testing as part of an ongoing surveillance activities in the laboratory; all isolates were de-identified prior to susceptibility testing.

*N. gonorrhoeae* isolates were identified from clinical specimens using standard laboratory protocols, including colony morphology, Gram stain, oxidase testing, and confirmation by enzymatic testing (Remel BactiCard Neisseria, ThermoFisher Scientific, Auckland, New Zealand). For antibiotic susceptibility testing, *N. gonorrhoeae* isolates were cultured using GC agar base supplemented with 1% isovitalex and incubated at 35-37^o^ C in 5% CO_2_. Antibiotic susceptibility testing was performed by disk diffusion method, using Oxoid antibiotic disks (Oxoid Limited, Basingstoke, UK) for penicillin, tetracycline, ciprofloxacin, spectinomycin, azithromycin, cefixime, and ceftriaxone according to the Clinical and Laboratory Standards Institute (CLSI) protocol [14]. CLSI interpretive criteria were used for penicillin, tetracycline, ciprofloxacin, cefixime, and ceftriaxone. In the absence CLSI-defined interpretive criteria for azithromycin by disk diffusion, interpretive criteria put forth by the CDC Neisseria Reference Laboratory were used, where susceptibility testing was performed using 15 μg disks and zone inhibition diameters ≤30 mm were defined as resistant, while those > 30 mm were not-resistant [15].

Quality control was performed using *N. gonorrhoeae* reference strains: ATCC 49226 and WHO P, G, and L strains [14, 16]. The laboratory participated in and passed external quality control assessments done by the WHO Gonococcal Antimicrobial Surveillance Program coordinated by the WHO Collaborating Centre for STD in Sydney, Australia, and the United Kingdom National External Quality Assessment Services (Sheffield, United Kingdom). Internal quality control was performed with each new lot of antibiotic discs or media, using reference strain ATCC 49226 [14].

The mean zone of inhibition diameters and corresponding standard deviations are reported. The mean zone of inhibition diameters for each antibiotic were compared by year using an Kruskal-Wallis test. Chi-square and Fisher’s exact tests were used to evaluate antibiotic susceptibility categories by year. In our data analysis, age was not normally distributed; we report median age and used a log-transformation of age for linear regression. Linear regression was used to evaluate trends in the zone of inhibition diameters for ceftriaxone, cefixime, and azithromycin by year, age, and sex. All data were analyzed using Stata 16 (Stata Corporation, College Station, TX, USA).

## Results

In total, there were 409 clinical isolates with antibiotic susceptibility data. The median age was 28 years, with a range from 16 to 70 years. Nearly all (88%) of the clinical specimens were obtained from males.

Mean zone of inhibition diameters and interpretative categories for each antibiotic are shown in Table 1. There were no isolates susceptible to penicillin and 402/409 (98.3%) of isolates were resistant to ciprofloxacin. All isolates were susceptible to spectinomycin.

**Table 1:** Antibiotic susceptibility data from 409 clinical isolates of *Neisseria gonorrhoeae* from Hanoi, Vietnam in 2017–2019. Mean zone diameters of inhibition and interpretive categories are presented for each antibiotic

**Antibiotics**	**Mean Zone of Inhibition Diameter, mm (SD)**			
	**2017** (*n* = 112)	**2018** (*n* = 135)	**2019** (*n* = 162)	***p*** value*
**Penicillin**	22.0 (11.9)	20.9 (10.2)	20.3 (10.5)	0.16
**Tetracycline**	22.8 (9.6)	21.7 (9.0)	24.6 (8.2)	< 0.01
**Ciprofloxacin**^**a**^	10 (6–10)	6 (6–16)	11 (6–17)	0.17
**Spectinomycin**	30.3 (3.3)	26.5 (3.3)	27.1 (3.7)	< 0.01
**Azithromycin**	34.6 (4.4)	31.6 (3.8)	32.7 (4.0)	< 0.01
**Ceftriaxone**	43.0 (4.1)	40.5 (3.6)	41.1 (3.8)	< 0.01
**Cefixime**	37.5 (5.0)	35.5 (4.3)	36.3 (4.1)	< 0.01
	**Interpretive Categories,**^**b**^ **n (%)**			
	**2017**	**2018**	**2019**	***p*** **value**
**Penicillin** (*n* = 408)				0.01
Susceptible	0 (0%)	0 (0%)	0 (0%)	
Intermediate	59 (52.7%)	50 (37.0%)	56 (34.6%)	
Resistant	53 (47.3%)	85 (63.0%)	106 (65.4%)	
**Tetracycline** (*n* = 409)				0.02
Susceptible	0 (0%)	1 (0.7%)	2 (1.2%)	
Intermediate	21 (18.8%)	12 (8.9%)	36 (22.2%)	
Resistant	91 (81.3%)	122 (90.4%)	124 (76.5%)	
**Ciprofloxacin** (*n* = 408)				0.24
Susceptible	0 (0%)	0 (0%)	2 (1.2%)	
Intermediate	0 (0%)	1 (0.7%)	3 (1.9%)	
Resistant	111 (100%)	134 (99.3%)	157 (96.9%)	
**Spectinomycin** (*n* = 409)				–
Susceptible	112 (100%)	135 (100%)	162 (100%)	
Intermediate	0 (0%)	0 (0%)	0 (0%)	
Resistant	0 (0%)	0 (0%)	0 (0%)	
**Azithromycin** (*n* = 407)				< 0.01
Non-resistant	94 (84.7%)	72 (53.3%)	119 (73.9%)	
Resistant	17 (15.3%)	63 (46.7%)	42 (26.1%)	
**Cefixime** (*n* = 406)				0.31
Susceptible	110 (98.2%)	125 (94.7%)	158 (97.5%)	
Non-susceptible	2 (1.8%)	7 (5.7%)	4 (2.5%)	
**Ceftriaxone** (*n* = 408)				0.78
Susceptible	112 (100%)	133 (99.3%)	160 (98.8%)	
Non-susceptible	0 (0%)	1 (0.7%)	2 (1.2%)	

*p value from Kruskal-Wallis test for means and Chi-square or Fisher’s Exact tests for susceptibility categories

^a^Median (Interquartile range)

^b^ Interpretive categories (except for azithromycin) were defined according to the Clinical and Laboratory Standards Institute (CLSI) protocol [14]. For azithromycin, interpretive criteria were defined according to the Centers for Disease Control Neisseria Reference Laboratory [15]

For azithromycin, the mean inhibition diameters were 34.6 mm in 2017, 31.6 mm in 2018, and 32.7 mm in 2019; the greatest difference in means was between years 2017 and 2018 (2.98 mm; 95% CI: 1.94, 4.01). In total, 122/407 (30.0%) isolates were resistant to azithromycin. There was an association between resistance and the year of collection (*p*-value < 0.01), ranging from 15.3% of isolates in 2017 to 46.7% of the isolates in 2018. For cefixime, the mean inhibition diameters were 37.5 mm in 2017, 35.5 mm in 2018, and 36.3 mm in 2019; the greatest difference in means was between years 2017 and 2018 (1.98 mm; 95% CI: 0.79, 3.17). Resistance to cefixime was found in 13/406 (3.2%) of isolates and there was no association by year (*p*-value 0.31). For ceftriaxone, the mean inhibition diameters were 43.0 mm in 2017, 40.5 mm in 2018, and 41.1 mm in 2019; the greatest difference in means was between years 2017 and 2018 (2.51 mm; 95% CI: 1.53, 3.49). Resistance to ceftriaxone occurred in 3/408 (0.7%) of isolates.

Results from univariate linear regression to predict zone of inhibition diameters by year, age, and sex for ceftriaxone, cefixime, and azithromycin are shown in Table 2. For ceftriaxone, diameters decreased by 0.83 mm each year (95% CI: − 1.3, − 0.4; *p* <  0.01), and decreased by 0.83 mm each year (95% CI: (− 1.33, − 0.33); *p* <  0.01) for azithromycin. There was no association between zone of inhibition diameters for cefixime by year (*p* = 0.07). Including age and sex in the multivariate linear regression models did not change the associations with year.

**Table 2:** Unadjusted and adjusted linear regression models to predict zone diameters of inhibition by year for ceftriaxone, cefixime, and azithromycin. The coefficient (β) is the slope of the regression line in mm

Antibiotic	Variable	Unadjusted		Adjusted	
		β (95% CI)	***p*** value	β (95% CI)	***p*** value
**Cefixime**	Year	- 0.50 (−1.04, 0.04)	0.069	−0.49 (−1.04, 0.05)	0.074
	Age	0.02 (−0.04, 0.08)	0.467	0.02 (−0.03, 0.08)	0.446
	Sex	0.34 (−1.02, 1.70)	0.624	0.25 (− 1.11, 1.62)	0.717
**Ceftriaxone**	Year	−0.83 (− 1.30, − 0.36)	0.001	− 0.83 (−91.30, − 0.35)	0.001
	Age	0.003 (− 0.05, 0.04)	0.90	−0.002 (− 0.05, 0.05)	0.93
	Sex	0.22 (−0.97, 1.41)	0.72	0.04 (−1.14, 1.22)	0.95
**Azithromycin**	Year	−0.83 (−1.33, − 0.33)	0.001	−0.81 (− 1.31, − 0.31)	0.002
	Age	0.12 (− 0.04, 0.06)	0.66	0.14 (− 0.04, 0.06)	0.59
	Sex	0.81 (−0.44, 2.06)	0.20	0.65 (−0.59, 1.90)	0.30

**Fig. 1:**
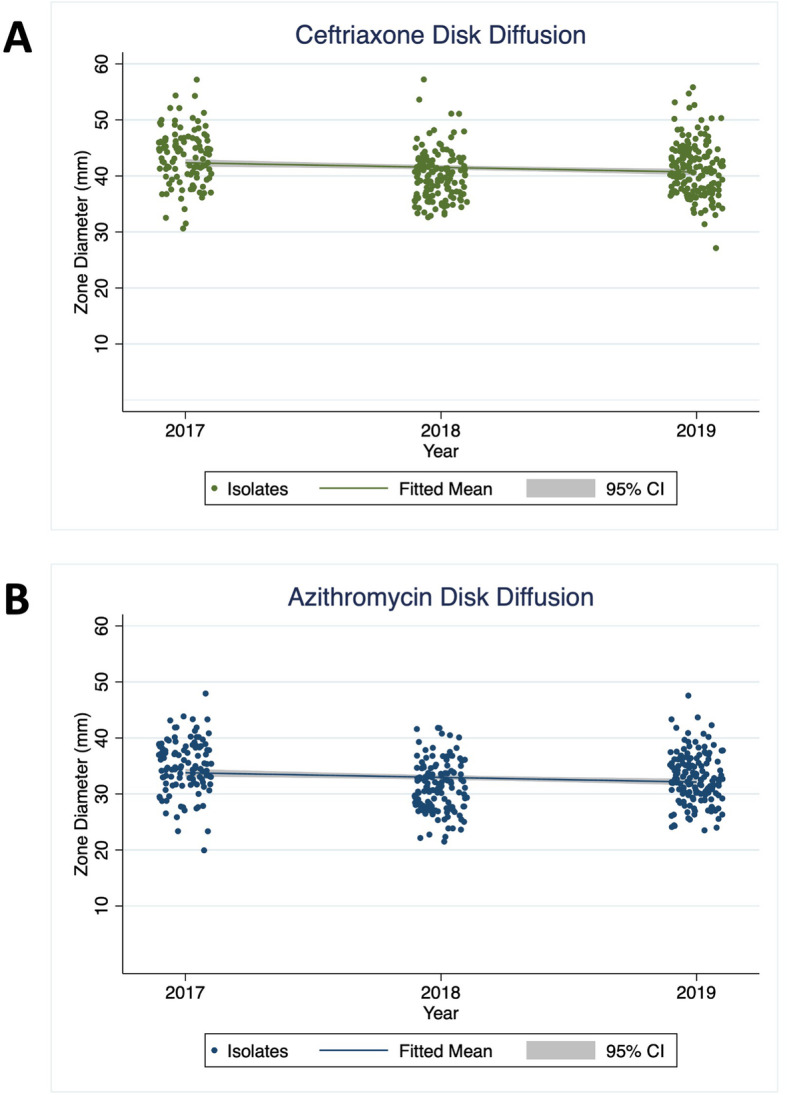
Scatter plot of disk diffusion data for *N. gonorrhoeae* isolates in Vietnam from 2017 to 2019. Zone diameters of inhibition in mm are shown, along with the fitted slope line and corresponding 95% confidence intervals (shaded gray), from the linear regression model. Panel **a** displays data for ceftriaxone and Panel **b** shows data for azithromycin

Scatter plots of disk diffusion data and the fitted means for azithromycin and ceftriaxone by year are shown in Fig. 1.

## Discussion

From 2017 to 2019, we found *N. gonorrhoeae* isolates exhibited decreasing trends in susceptibility to azithromycin and ceftriaxone each year. While few isolates were resistant to ceftriaxone or cefixime, we identified a high prevalence of resistance to azithromycin. The trends of decreasing susceptibilities to both first-line treatment agents are concerning, underscoring the urgency of addressing AMR in *N. gonorrhoeae* and the need for ongoing surveillance in the Western Pacific Region.

Our report provides updated data in antibiotic susceptibility from Vietnam. The most recent WHO-GASP report, which included 2011–2016 isolates, found < 5% were resistant to azithromycin, but ≥5% were resistant to ceftriaxone, although only ceftriaxone data from 2015 were reported [11]. A report by Lan et al. on isolates from 2015 to 2016 in Vietnam identified a low prevalence (1%) of ceftriaxone-resistant strains, similar to our findings; however, they reported resistance to cefixime in 15% of isolates, compared to 3% in our report [12]. That report noted a trend towards decreasing susceptibility to azithromycin, but found 5% of isolates were resistant to azithromycin [12]. While we provide data on more recent *N. gonorrhoeae* isolates, different sampling populations or different interpretive criteria might contribute to the observed differences in susceptibility. Those reports, including our own, do not consist of systematic sampling of isolates, thus are somewhat limited in their generalizability. Nevertheless, they contribute important data regarding antibiotic susceptibility of *N. gonorrhoeae* in Vietnam.

In our report, resistance to penicillin, tetracycline, and ciprofloxacin were all high, similar to other reports from the region and supporting the recommendations that these agents should not be used for treatment [12, 17]. Our report suggests that the WHO’s historical 5% resistance threshold might be surpassed for azithromycin in Vietnam, similar to other countries in the WPR [11]. Interestingly, all isolates were susceptible to spectinomycin, as were those from Lan et al. [12], suggesting spectinomycin might have a limited role in treatment of uncomplicated urethral infections, although there remain significant limitations to its use, including limited availability, poor treatment of pharyngeal infections, and the low barrier to resistance [4]. Our data support the continued use of ceftriaxone for gonorrhea treatment in Vietnam, but continued monitoring of susceptibility trends is needed.

Our limitations include that data were from one hospital in Hanoi and might not be representative of other locations or settings in the country. We report antibiotic susceptibilities using disk diffusion according to CLSI where available; however, in the absence of CLSI-defined interpretation for azithromycin, we used CDC Neisseria Reference Laboratory interpretive criteria for disk diffusion. Lastly, we did not have epidemiologic or clinical characteristics of the isolates and thus were unable to assess risk factors for resistance. As such, it was not possible to determine if increased sampling over time occurred from populations (e.g.- men who have sex with men or commercial sex workers) or type of infections (e.g.- test-of-cure, persistent, or pharyngeal infections) at higher risk for antimicrobial resistance, which could have contributed to our observed results.

## Conclusions

We report decreasing susceptibility of *N. gonorrhoeae* to ceftriaxone and azithromycin, as well as a high prevalence of resistance to azithromycin from Hanoi, Vietnam in 2017–2019. The trends of decreasing susceptibility to first-line treatments are concerning and highlight the urgency of addressing antimicrobial resistance in *N. gonorrhoeae*. Expanded surveillance efforts within the WPR will be critical to monitoring trends and informing treatment guidelines.

## Data Availability

The data will be shared upon reasonable request made to the corresponding author.

## Abbreviations

AMR: Antimicrobial resistance
CLSI: Clinical and Laboratory Standards Institute
WHO: World Health Organization
CDC: U.S. Centers for Disease Control and Prevention
WPR: Western Pacific Region
GASP: Gonococcal Antimicrobial Surveillance Programme
